# Dynein Associates with *oskar* mRNPs and Is Required For Their Efficient Net Plus-End Localization in *Drosophila* Oocytes

**DOI:** 10.1371/journal.pone.0080605

**Published:** 2013-11-11

**Authors:** Paulomi Sanghavi, Shobha Laxani, Xuan Li, Simon L. Bullock, Graydon B. Gonsalvez

**Affiliations:** 1 Cellular Biology and Anatomy, Georgia Regents University, Augusta, Georgia, United States of America; 2 Division of Cell Biology, Medical Research Council Laboratory of Molecular Biology, Cambridge, United Kingdom; Biogen Idec, United States of America

## Abstract

In order for eukaryotic cells to function properly, they must establish polarity. The *Drosophila* oocyte uses mRNA localization to establish polarity and hence provides a genetically tractable model in which to study this process. The spatial restriction of *oskar* mRNA and its subsequent protein product is necessary for embryonic patterning. The localization of *oskar* mRNA requires microtubules and microtubule-based motor proteins. Null mutants in Kinesin heavy chain (Khc), the motor subunit of the plus end-directed Kinesin-1, result in *oskar* mRNA delocalization. Although the majority of *oskar* particles are non-motile in khc nulls, a small fraction of particles display active motility. Thus, a motor other than Kinesin-1 could conceivably also participate in *oskar* mRNA localization. Here we show that Dynein heavy chain (Dhc), the motor subunit of the minus end-directed Dynein complex, extensively co-localizes with Khc and *oskar* mRNA. In addition, immunoprecipitation of the Dynein complex specifically co-precipitated *oskar* mRNA and Khc. Lastly, germline-specific depletion of Dhc resulted in *oskar* mRNA and Khc delocalization. Our results therefore suggest that efficient posterior localization of *oskar* mRNA requires the concerted activities of both Dynein and Kinesin-1.

## Introduction

Many cellular processes such as endocytosis, cell division, and cell migration require the specific and active transport of molecules to defined cellular sites [1]. This transport is commonly mediated by motor proteins, a class of proteins that use the energy derived from ATP hydrolysis to move cargo within the cell. Motor proteins transport cargo on microtubule or actin based cytoskeletal structures. In general, long-range transport is carried out by microtubule-based motors, whereas short-range transport is mediated by motors that traverse the actin cytoskeleton [1].

Most members of the Kinesin family of motor proteins transport cargo toward the plus end of microtubules, whereas cytoplasmic Dynein delivers cargo to the microtubule minus end [2,3]. Over the past two decades, in vitro reconstitution of motor-based transport has revealed important mechanical properties of conventional Kinesin (Kinesin-1) and Dynein [4-6]. However, the mechanism by which these motors function in a complex cellular environment to transport specific cargoes remains relatively unknown.

The *Drosophila* oocyte is an excellent model in which to examine the in vivo function and regulation of microtubule motors. Within the oocyte, *oskar* mRNA is specifically localized at the posterior pole [7,8]. This localization pattern, coupled with translational repression of unlocalized transcripts, results in restriction of Oskar protein to the oocyte posterior [9-11]. Oskar protein functions to establish the anterior-posterior polarity of the oocyte and resulting embryo [12,13]. Oskar protein is also required for germ cell specification during embryogenesis [12,13]. In addition to *oskar*, *bicoid* mRNA localizes to the anterior margin of the oocyte and *gurken* mRNA localizes to the dorsal-anterior corner [14,15]. As with *oskar*, the spatial restriction of these mRNAs and subsequent protein products is essential for establishing the polarity of the oocyte and future embryo [16,17]. 

The posterior localization of *oskar* mRNA is dependent on microtubules and Kinesin heavy chain (Khc), the motor subunit of the Kinesin-1 complex [2,18,19]. The involvement of Khc in the posterior transport of *oskar* mRNA is supported by several lines of evidence. Khc and *oskar* mRNA co-localize at the posterior of the oocyte [20,21]. Khc is a plus end-directed motor and the posterior of the oocyte is enriched for microtubules plus ends [2,22,23]. *oskar* mRNA is delocalized in khc null oocytes and live imaging revealed that the majority of oskar mRNP particles are non motile in the absence of Khc [18,24]. However, a small percentage of *oskar* mRNP particles displayed active and directional motility in khc null oocytes [24]. This finding suggests the involvement of an additional, unidentified motor in the transport of *oskar* mRNA. 

In addition to Khc and *oskar* mRNA, Dynein heavy chain (Dhc), the motor sub-unit of Dynein complex, also localizes at the oocyte posterior [25]. This finding was unexpected because Dynein is a minus end-directed motor [3]. However, recent evidence suggests that opposite polarity motors often coordinate their activities to transport cargo within the cell [26]. For instance, transport of fmr1 mRNA requires Khc and Dhc [27]. In addition, the bi-directional motility of lipid droplets in *Drosophila* blastoderm embryos, and peroxisomes in cultured *Drosophila* cells, also requires the activities of both motor proteins [28,29]. We therefore hypothesized that Dynein might function in the localization of *oskar* mRNA, and as a consequence of this function, the motor complex is enriched at the posterior pole. The goal of the present study was to test this hypothesis. In support of this hypothesis, our findings indicate that Dynein is present in a complex with *oskar* mRNA in vivo. In addition, we demonstrate that efficient posterior localization of *oskar* mRNA and Khc requires Dynein.

## Results

### The Dynein complex is associated with *oskar* mRNA in vivo

Previous studies suggest a pivotal role for Kinesin-1 in the transport of *oskar* mRNA [18]. We postulated that in addition to Kinesin-1, Dynein might also be involved. However, to date, neither motor has been shown to associate with *oskar* mRNA in vivo. In order to determine which motors are present in a complex with *oskar* mRNA, we immunoprecipitated Khc, Dhc, Glued/p150 and Lis-1 from wild-type lysates. Glued is the largest subunit of the Dynactin complex, a major regulator of Dynein [3]. Lis-1 is thought to regulate the activity of Dynein by binding to the catalytic domain of the motor [3]. RNA was extracted from the immunoprecipitates and the presence of *oskar* mRNA was analyzed using RT-PCR. 

**Figure 1:**
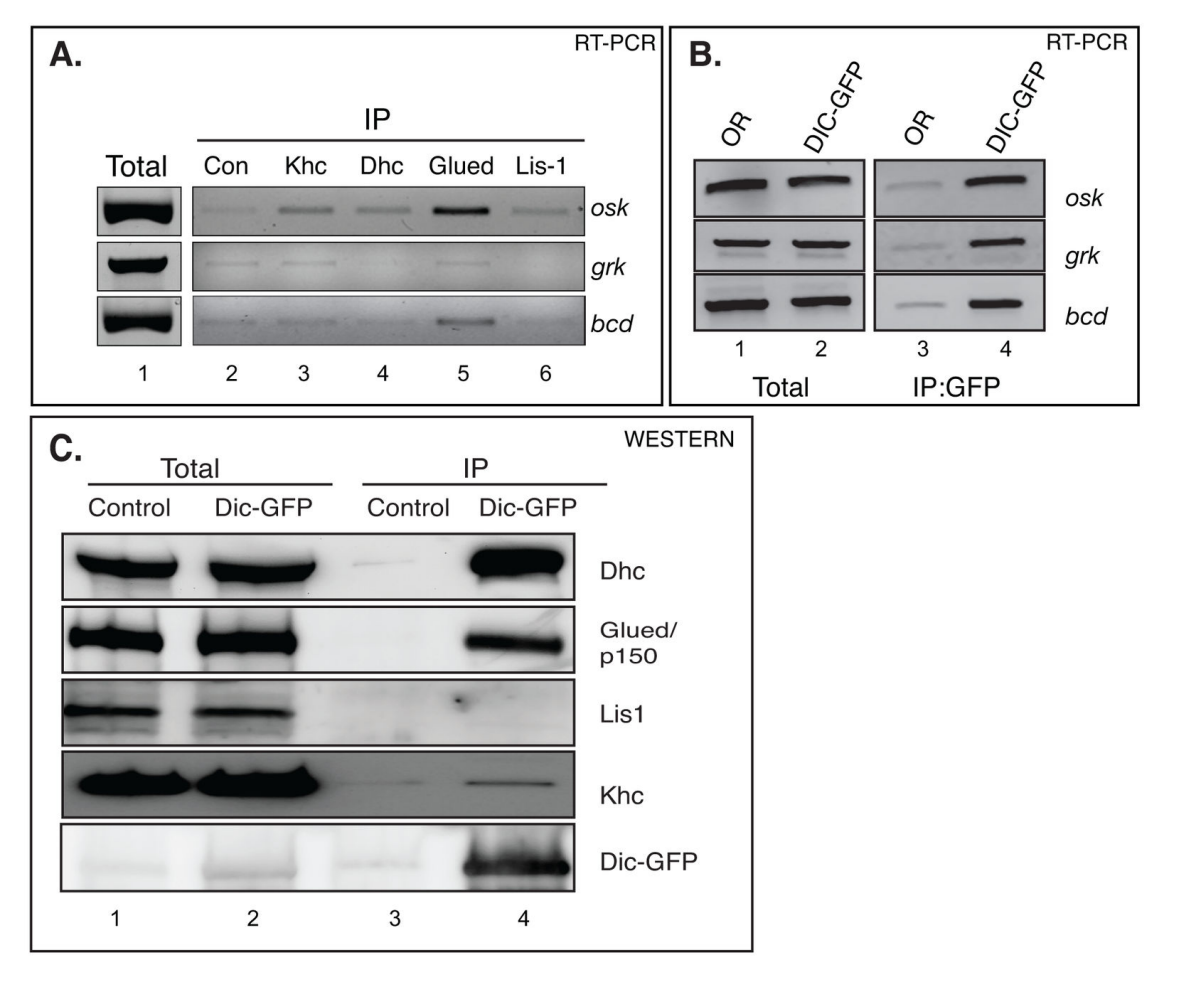
The Dynein complex associates with *oskar* mRNA in vivo. (A) Ovarian lysates from wild-type flies were subjected to immunoprecipitation using the following antibodies; a goat anti-mouse control antibody (lane 2), Khc (lane 3), Dhc (lane 4), Glued/p150 (lane 5), and Lis-1 (lane 6). The co-precipitating RNAs were extracted and analyzed using RT-PCR. A specific enrichment of *oskar* mRNA could be detected in the Glued pellet. A small but significant enrichment of *bicoid* mRNA could also be detected in the Glued pellet. Lane 1 represents total RNA from wild-type flies analyzed using primers against *oskar*, *bicoid* and *gurken*. This experiment was repeated three times. (B) Ovarian lysates from wild-type flies and flies expressing Dic-GFP were subjected to immunoprecipitation using GFP antibody beads. The co-precipitating RNAs were extracted and analyzed as in panel A (lanes 3 and 4). *oskar*, *bicoid* and *gurken* mRNAs were specifically enriched in the Dic-GFP pellet. Total RNA from the same flies was also analyzing using RT-PCR with primers against *oskar*, *bicoid* and *gurken* (lanes 1 and 2). The IP RT-PCR experiment was repeated four times. (C) Ovarian lysates from wild-type flies and flies expressing Dic-GFP were subjected to immunoprecipitation using GFP antibody beads. The precipitates were run on gel and analyzed by western blotting using the indicated antibodies (lanes 3 and 4). The total fraction from both lysates were similarly analyzed (lanes 1 and 2). As expected, Dhc and Glued co-precipitated with Dic-GFP. In addition, a small enrichment of Khc was also detected in the Dic-GFP pellet. This experiment was repeated three times.

Using this strategy, we observed a specific enrichment of *oskar* and *bicoid* mRNAs in the Glued pellet (Figure 1A). However, antibodies against Khc and Dhc did not efficiently precipitate any of the tested mRNAs (Figure 1A). One explanation for this result is that neither Kinesin-1 nor Dynein are associated with *oskar* mRNA. An alternative explanation is that antibodies against Khc and Dhc do not efficiently precipitate the motor/cargo complexes. In order to test the latter possibility, we prepared lysates from flies expressing Dynein intermediate chain (Dic) tagged with GFP (Dic-GFP). This enabled us to purify the Dynein complex using GFP antibody beads. RNA was extracted from the immunoprecipitates and analyzed using RT-PCR. *oskar* mRNA was significantly enriched in the Dic-GFP pellet (Figure 1B). Based on these results, we conclude that the Dynein motor is present in a complex with *oskar* mRNA in vivo. In addition to *oskar*, *bicoid* and *gurken* mRNAs were also detected in the Dic-GFP pellet (Figure 1B). These results are consistent with published findings suggesting the involvement of the Dynein motor in the anterior localization of *bicoid* and *gurken* mRNAs [20,30-34]. In contrast to these mRNAs, non-localized mRNAs such as *smb* and *vasa* were present at background level in control and Dic-GFP pellet (Figure S1).

Although the Dynactin complex and Lis-1 are both thought to regulate Dynein, the mechanism by which they function is unclear. Our finding that antibodies against Glued co-precipitated *oskar* mRNA suggests that the Dynactin complex might be stably associated with Dynein in vivo. In order to test this prediction, we immunoprecipitated Dic-GFP from ovarian lysates. The co-precipitating proteins were then analyzed by western blotting. As expected, Dhc significantly co-precipitated with Dic-GFP (Figure 1C). In addition, Glued was also enriched in the Dic-GFP pellet (Figure 1C). By contrast, we could not detect Lis-1 in the same pellet (Figure 1C). Thus, the Dynactin complex, but not Lis-1, is stably associated with Dynein in vivo. A small amount of Khc was also detected in the Dic-GFP pellet (Figure 1C). This raised the possibility that Khc, Dynein and *oskar* mRNA might be present in a complex in vivo.

**Figure 2:**
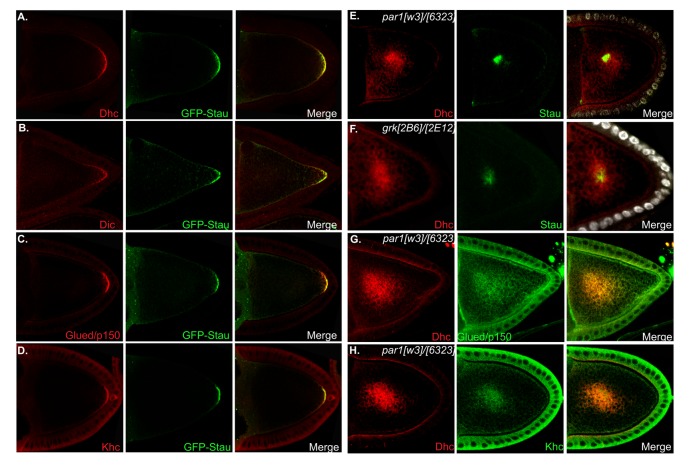
The Dynein complex co-localizes with *oskar* mRNA in wild-type and polarity-defective oocytes. (A-D) Ovaries were dissected from wild-type flies expressing GFP-Staufen. The egg chambers were stained with antibodies against Dhc (A), Dic (B), Glued/p150 (C) and Khc (D). In order to boost the GFP fluorescence which typically fades during the 3 day procedure, the samples were also incubated with either a rabbit anti-GFP antibody (A and B) or a rat anti-GFP antibody (C and D). Note that Dhc, Dic, Glued and Khc co-localized with GFP-Staufen at the oocyte posterior. (E-H) *par-1* mutant ovaries were dissected and stained with antibodies against Dhc and Staufen (E), Dhc and Glued (G) and Dhc and Khc (H). Ovaries that were mutant for *gurken* were dissected and stained with antibodies against Dhc and Staufen (F). The Dynein complex and Khc localized in a central focus in these mutant egg chambers.

In order to test this possibility, we examined the localization of motor complex components in wild-type and mutant oocytes. Khc, Dhc, Dic and Glued co-localized with GFP-Staufen in stage 9 and 10a wild-type oocytes (Figures 2A-2D, and data not shown). GFP-Staufen serves as a faithful reporter for the localization of *oskar* mRNA due to Staufen’s role as a core component of the *oskar* RNP [24]. We next examined the localization of Dynein in *par-1* and *gurken* mutant oocytes. Mutations in these genes result in polarity defects within the oocyte [35-38], causing *oskar* mRNA and proteins associated with this transcript to localize to the center of the oocyte [39-41]. Dhc partially co-localized with Staufen in the center of *par-1* and *gurken* mutant oocytes (Figures 2E and 2F). A similar result was also obtained for Glued and Khc (Figures 2G, 2H and data not shown). It should be noted that the central foci observed for Dhc, Glued and Khc were more diffuse that the one observed for Staufen.

Collectively, these results suggest that Dynein and Khc are present in a complex with *oskar* mRNA. However, we cannot conclude the precise nature or stoichiometry of this complex. For instance, these data do not distinguish between a scenario in which Dynein and Kinesin are present in separate complexes with *oskar* mRNA, versus a scenario in which both motors are present together in the same complex with *oskar* mRNA. 

### Depletion of Dhc results in *oskar* mRNA delocalization

We next determined whether Dynein was required for *oskar* mRNA localization. Previous attempts at analyzing the function of Dynein in mature oocytes were hampered by the requirement of this motor in oocyte specification. Loss-of-function mutants in *dhc* fail to specify an oocyte and the available, weak hypomorphic mutants show no overt defects in steady-state mRNA localization [42]. In order to overcome this limitation, we depleted Dhc in mid-stage egg chambers using a newly described shRNA approach [43]. ShRNAs targeting different regions of *dhc* mRNA were transcribed using a driver that is expressed are very low levels in early-stage egg chambers, but is active during mid to late oogenesis (maternal alpha-tubulin-Gal4; see Materials and Methods for details) (Figure S2). This enabled us to bypass the requirement of Dynein in oocyte specification.

**Figure 3:**
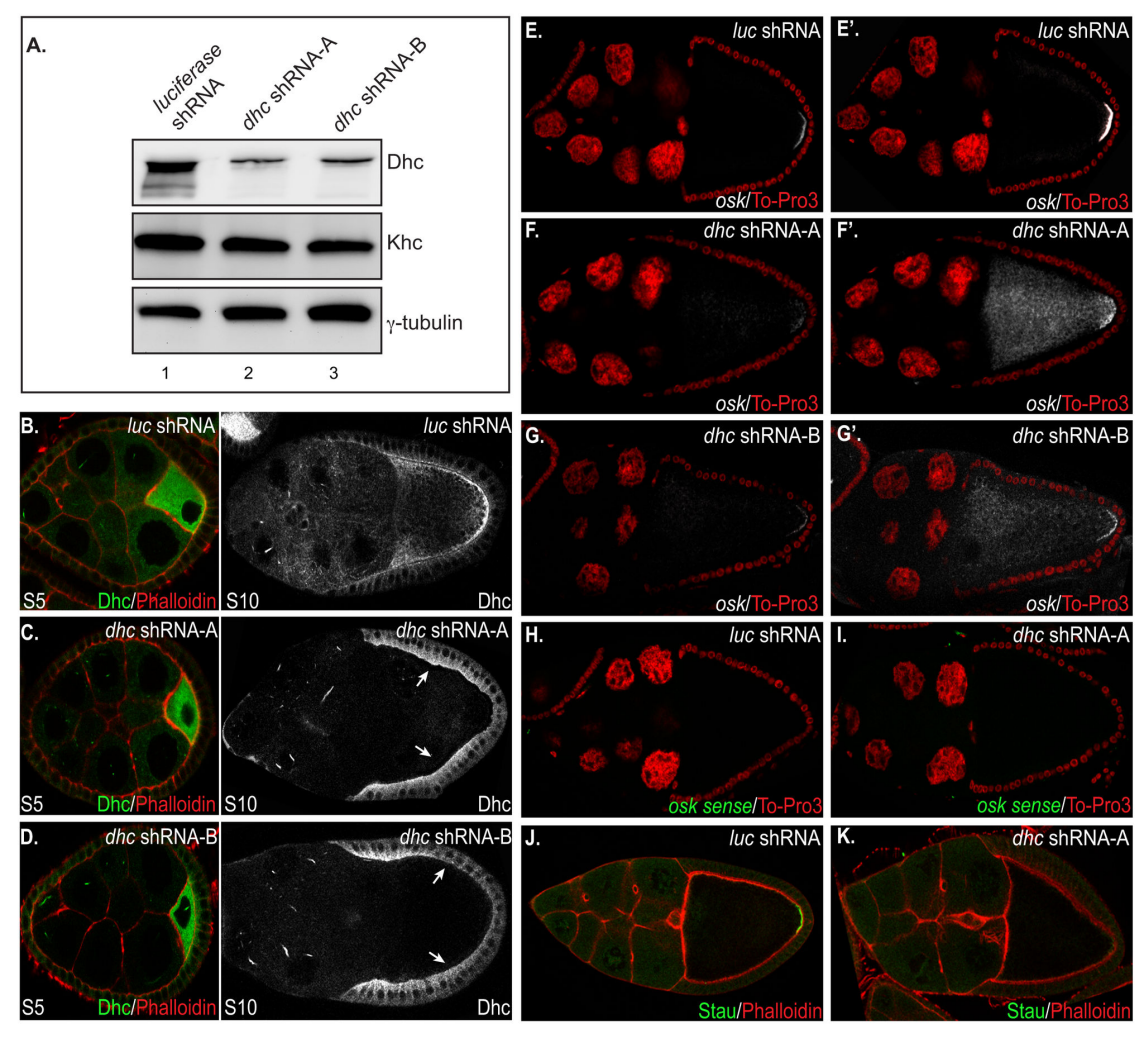
Dynein is required for posterior *oskar* mRNA localization. (A) Ovarian lysates were prepared from flies expressing shRNAs against *luciferase* (lane 1) and *dhc* (lanes 2 and 3). The lysates were run on a gel and analyzed by western blotting using the indicated antibodies. Dhc levels were dramatically reduced in *dhc* shRNA expressing lysates. By contrast, Khc and gamma-tubulin levels were unaffected. This experiment was repeated three times. (B-D) Ovaries were dissected from flies expressing shRNAs against *luciferase* (B) and *dhc* (C, D). The egg chambers were stained with an antibody against Dhc and were counterstained with TRITC-conjugated Phalloidin (actin stain). The panels on the left depict early-stage egg chambers (S5), whereas those on the right are mid-stage egg chambers (S10). Note that whereas Dhc levels were the same in early-stage egg chambers, the level of Dhc was greatly reduced in the germline of *dhc* shRNA expressing mid-stage egg chambers. The arrows indicate the strong signal for Dhc in the overlying somatic follicle cells. Dhc depletion was verified using immunofluorescence on more than ten occasions. (E-G) Ovaries were dissected from flies expressing shRNAs against *luciferase* (E) and *dhc* (F,G). The egg chambers were processed for in situ hybridization using anti-sense probes against *oskar* mRNA. The egg chambers were imaged under conditions of low-gain (E, F and G) and high-gain (E’, F’ and G’). Expression of shRNA targeting *luciferase* had no effect on the localization of *oskar* mRNA. By contrast, depletion of Dhc resulted in significant *oskar* mRNA delocalization. *oskar* mRNA was delocalized in 82% of egg chambers expressing *dhc* shRNA-A (n=191) and in 87% (n=106) of egg chambers expressing shRNA-B. (H-I) In order to validate the *oskar* mRNA in situ signal, control (H) and Dhc depleted (I) ovaries were dissected and processed for in situ hybridization using sense probes against *oskar* mRNA. Ten times more sense probe was used in this experiment in comparison to the anti-sense probes used in panels E-G. Despite the abundance of probe, no in situ signal was observed. (J-K) Egg chambers from flies expressing shRNAs targeting *luciferase* (J) and *dhc* (K) were stained with an antibody against Staufen. The egg chambers were also counterstained with Alexa 633-conjugated Phalloidin. Staufen, which serves as a marker for *oskar* mRNA, is localized at the posterior pole in control oocytes. In contrast, Dynein depletion results in greatly reduced posterior Staufen (73% affected; n=130).

In order to test the efficacy of Dynein depletion, we prepared ovarian lysates from flies expressing shRNA against *luciferase* (negative control), and from flies expressing shRNAs targeting different regions of *dhc* (*dhc* shRNA-A and B). The lysates were run on a gel and analyzed by western blotting (Figure 3A). In comparison to the control, the level of Dhc was significantly depleted upon expression of *dhc* shRNAs (Figure 3A). By contrast, the level of Khc and gamma-tubulin were the same in control and Dhc depleted lysates (Figure 3A). Thus, expression of shRNAs targeting *dhc* resulted in specific depletion of the corresponding protein.

In order to determine the specificity of Dhc depletion, we performed immunofluorescence on dissected ovaries. As expected, expression of shRNA targeting *luciferase* had no effect on the level and localization of Dhc (Figure 3B). By contrast, expression of either *dhc* shRNA-A or B resulted in significant depletion of Dhc in the germline of mid-stage egg chambers (Figures 3C and 3D). Consistent with the expression profile of the driver, Dhc levels were unaffected in early-stage egg chambers (Figures 3B-D; left panels) and in the overlying somatic follicle cells of mid-stage egg chambers (Figure 3B-D; right panel, arrows).

Ovaries from flies expressing shRNA against *luciferase* or *dhc* were next processed for in situ hybridization using anti-sense probes against *oskar* mRNA. As expected, *oskar* mRNA was localized in a tight crescent at the posterior of oocytes expressing shRNA against *luciferase* (Figure 3E). When imaged using confocal microscopy under these same non-saturating conditions, very little *oskar* mRNA could be detected at the posterior pole in Dhc depleted egg chambers (Figures 3F and 3G). Because the level of *oskar* mRNA was not affected by Dhc depletion (Figure S3), this result suggests that *oskar* mRNA might be delocalized in the depleted egg chambers. In order to visualize the delocalized mRNA, we imaged the egg chambers under conditions of higher gain, the confocal equivalent of a “longer exposure”. Under these conditions, the bulk of *oskar* mRNA in the Dhc depleted egg chambers was delocalized in the oocyte cytoplasm (Figures 3F’ and 3G’). By contrast, under the same conditions, the posterior signal in the control oocyte was saturated, yet very little delocalized mRNA could be detected (Figure 3E’). In order to verify the specificity of the *oskar* mRNA in situ signal, we processed control and Dhc depleted egg chambers with sense-probe against *oskar* mRNA. Even though we used ten times more sense probe than anti-sense, and imaged under the same conditions of high gain, no in situ signal was obtained (Figures 3H and 3I). We therefore conclude that depletion of Dhc results in significant delocalization of *oskar* mRNA.

In order to validate the requirement of Dhc in the localization of *oskar* mRNA, we examined the localization of Staufen, a core component of the *oskar* mRNP. Consistent with the results obtained using in situ hybridization, Staufen localized in a tight posterior crescent in *luciferase* shRNA expressing oocytes (Figure 3J). By contrast, only a residual amount of posterior Staufen was detected in Dhc depleted oocytes (Figures 3K) (73% affected). 

This strategy of shRNA-mediated depletion has not been extensively used to study processes during mid-oogenesis. We therefore included an additional control to demonstrate the specificity of the Dhc depletion phenotype. Ovaries were dissected and processed from flies expressing shRNA against *luciferase* or *eb1*. EB1 is a microtubule plus end binding protein that is abundantly expressed in the *Drosophila* germline [23,44]. Expressing shRNA targeting *eb1* successfully depleted EB1, yet had no effect on the localization of either Dhc or *oskar* mRNA (Figure S4). 

### Effects of Dhc depletion on Oskar protein levels

**Figure 4:**
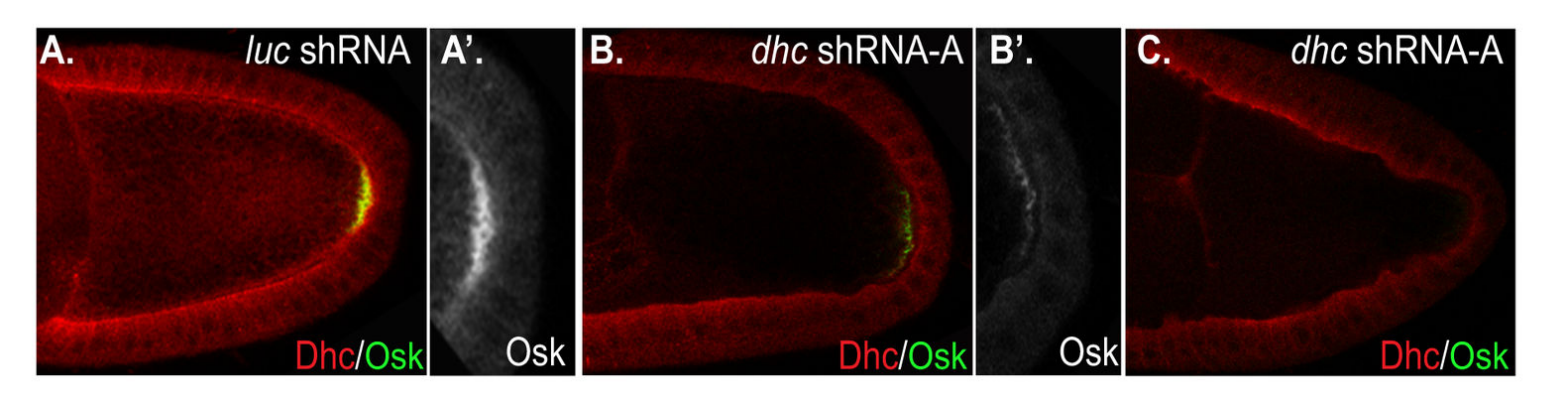
Oskar protein levels are reduced in Dhc depleted egg chambers. Egg chambers from flies expressing shRNAs targeting *luciferase* (A) and *dhc* (B, C) were stained with antibodies against Dhc (Red) and Oskar protein (Green and grey scale). Oskar protein is abundant at the posterior pole in control egg chambers. However, Dynein depletion results in greatly reduced (59%), or absent (7%) posterior Oskar protein (n=84). A’ and B’ are magnified views of the oocyte posterior in which Oskar is shown in grey scale.

We next examined the localization and level of Oskar protein. The translation of *oskar* mRNA is repressed during transit. Only once *oskar* mRNA is localized at the posterior pole is repression relieved and the mRNA activated for translation [45]. Consistent with the in situ hybridization result, Oskar protein was abundantly localized at the posterior pole in control oocytes (Figure 4A). By contrast, most egg chambers that were depleted of Dhc displayed greatly reduced levels of posterior Oskar protein (Figures 4B and 4C). The level of Oskar protein corresponded to the residual amount of Dhc in these oocytes. If trace amounts of Dhc could be detected, a small amount of posterior Oskar was present (Figure 4B). However, if Dhc was undetectable in mid-stage oocytes, very little if any Oskar protein was observed (Figure 4C).

Based on these results, we conclude that Dhc is required for efficient posterior localization of *oskar* mRNA. Depletion of Dhc results in delocalization of *oskar* mRNA, and as a consequence, a corresponding reduction in the amount of Oskar protein.

### Additional functions of Dynein in mid-stage egg chambers

**Figure 5:**
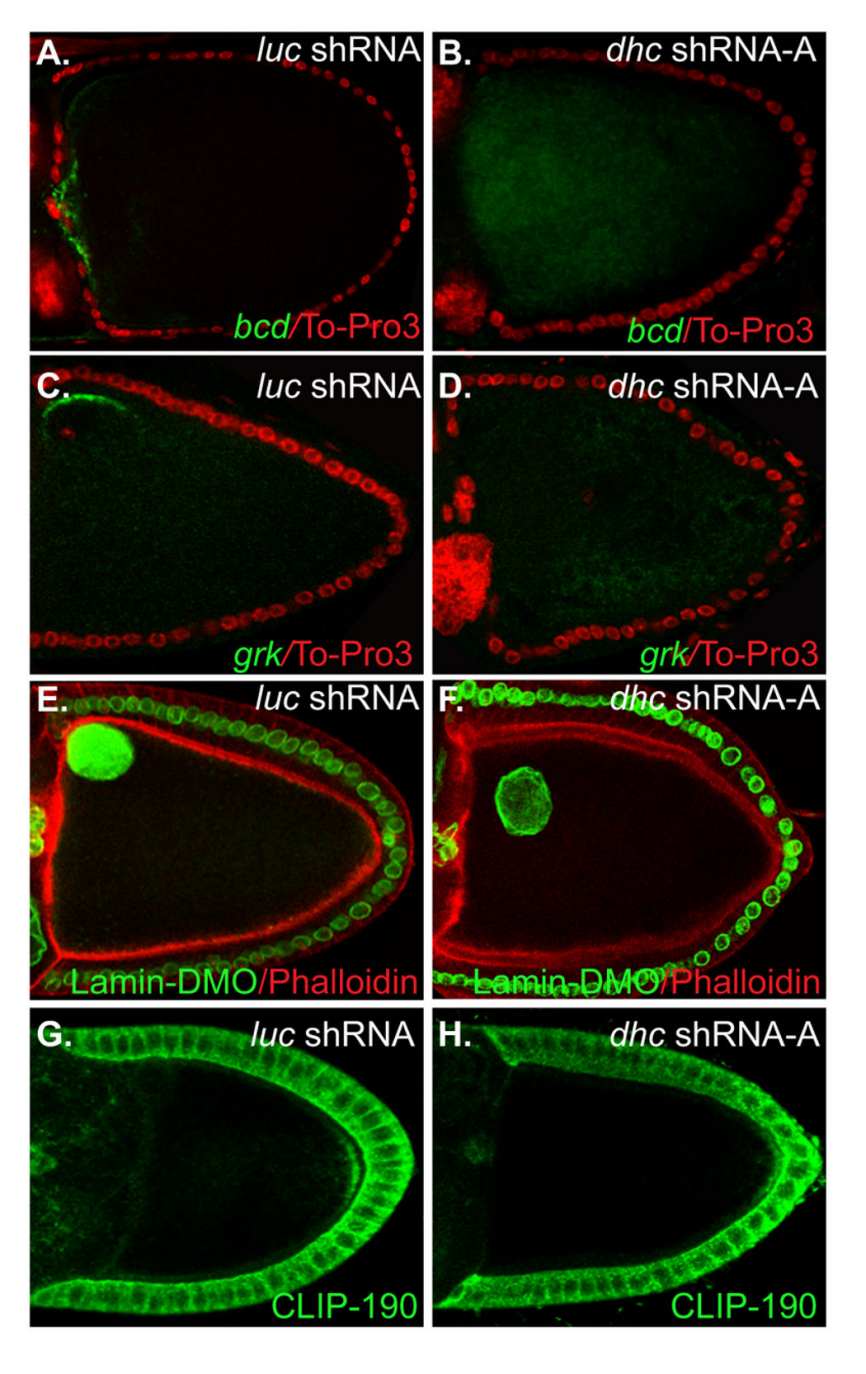
Additional germline function of Dynein. (A-D) Egg chambers from flies expressing control and *dhc* shRNAs were processed for in situ hybridization using anti-sense probes against *bicoid* (A, B) and *gurken* mRNA (C,D). *bicoid* and *gurken* mRNAs were correctly localized in control oocytes. However, both transcripts were delocalized in strains expressing an shRNA targeting *dhc*. 78% of egg chambers contained delocalized *bicoid* mRNA (n=51). 73% of egg chambers contained delocalized *gurken* mRNA (n=33). (E-F) The oocyte nucleus was visualized in control (E) and Dhc depleted oocytes (F) using an antibody against Lamin DmO. The oocyte nucleus was anchored at the dorsal anterior cortex of control oocytes. The anchoring of the oocyte nucleus was disrupted in approximately 10% of Dhc depleted egg chambers. (G-H) Microtubule polarity was determined by immunostaining control and Dhc depleted oocytes using an antibody against the plus-end marker protein, CLIP-190. 98% of egg chambers expressing shRNA against *luciferase* has posterior localized CLIP-190 (n=64 egg chambers). By contrast, 84% of egg chambers expressing shRNA against *dhc* had a reduced level of posterior CLIP-190, 13% of egg chambers had no detectable posterior CLIP-190, and 3% displayed a pattern that was undistinguishable from the control (n=69 egg chambers).

Previous studies have implicated a function for Dynein in the anterior localization of *bicoid* and *gurken* mRNAs [20,30-34]. In the case of endogenous *bicoid* and *gurken* mRNAs, this was demonstrated by over-expressing the p50/Dynamitin subunit of the Dynactin complex [30,32,34]. This complex is known to regulate the activity of Dynein and over-expression of Dynamitin causes the Dynactin complex to dissociate [46]. Thus, defects observed upon Dynamitin over-expression are inferred to result from compromised Dynein activity. We therefore chose to directly address the requirement of Dynein in the anterior localization of *bicoid* and *gurken* mRNAs by specifically depleting Dhc in stage 10 egg chambers. In contrast to oocytes expressing shRNA targeting luciferase, *bicoid* and *gurken* mRNAs were significantly delocalized in Dhc depleted oocytes (Figures 5A-D; 78% and 73% respectively). Thus, Dynein is required for the anterior localization of these cargoes.

Over-expression of Dynamitin causes detachment of the oocyte nucleus form the anterior cortex [30,32]. A similar phenotype is also observed in lis-1 mutants [47,48]. These results suggest that Dynein might be required for anchoring the oocyte nucleus. In order to test this prediction, we depleted Dhc in mid-stage egg chambers. Consistent with previous studies, Dhc depletion resulted in displacement of the oocyte nucleus from the anterior cortex (Figures 5E and 5F).

The defective localization of *oskar*, *bicoid*, and *gurken* mRNAs, and the detachment of the oocyte nucleus from the anterior cortex, is unlikely to be caused by aberrant polarization of the microtubule cytoskeleton. CLIP-190, a marker for microtubule plus-ends, was localized at the posterior pole in most Dhc depleted egg chambers (Figures 5G and 5H). However, the level of posterior CLIP-190 was often reduced in comparison to the control (84%; n=69). Because Oskar protein is required for recruiting microtubule plus ends to the posterior pole [23,49], the deficit of posterior CLIP-190 likely reflects an upstream reduction in Oskar protein levels (Figures 4B and 4C). The gross organization of alpha-tubulin was the same in control and Dhc depleted oocytes (data not shown). In addition, the anterior migration of the oocyte nucleus, a process that requires polarized and dynamic microtubules [50], occurred normally in Dhc depleted oocytes (Figure 5F). Lastly, we observed no focal accumulations of *oskar* mRNA, as has been described in polarity-defective mutants [37,38]. Collectively, these results suggest that microtubules are normally polarized under conditions of Dhc depletion. 

### The posterior localization of Khc and Dynactin require Dynein

**Figure 6:**
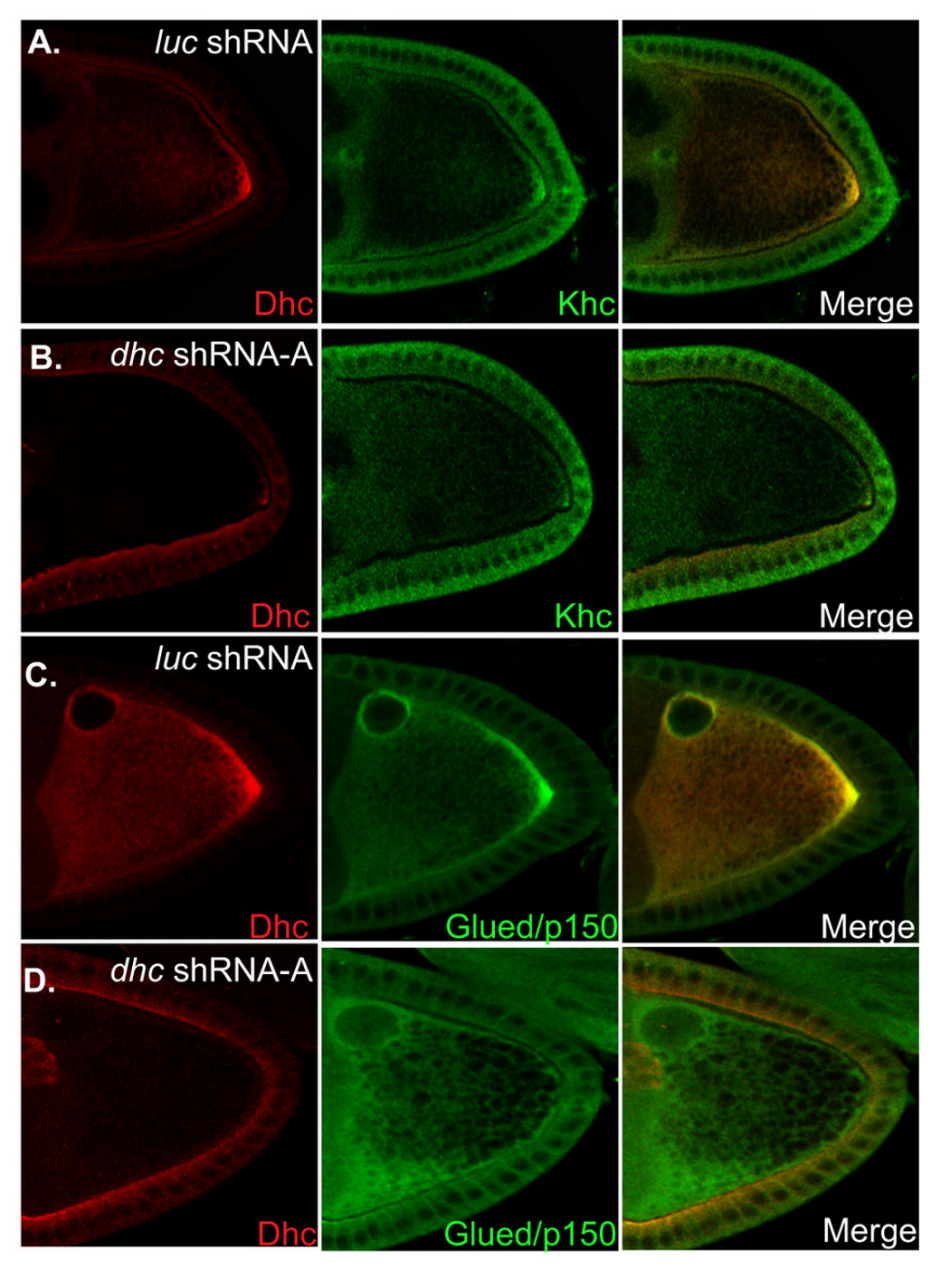
The posterior localization of Khc and Glued requires Dynein. (A-B) Egg chambers from control and *dhc* shRNA expressing flies were immunostained using antibodies against Dhc (Red) and Khc (Green). Whereas Khc was enriched at the posterior pole in control oocytes, the posterior accumulation of Khc was greatly reduced in Dhc depleted oocytes. (C-D) The same strains were also processed for immunofluorescence using antibodies against Dhc (Red) and Glued (Green). As with Khc, the posterior accumulation of Glued was reduced in Dhc depleted oocytes in comparison to the control.

The available evidence suggests that Khc is the primary motor involved in the net plus-end localization of *oskar* mRNA [18,24]. Depletion of Dhc has no effect on the total level of Khc, yet the posterior localization of *oskar* mRNA is significantly affected (Figure 3). One possible explanation for this phenotype is that when Dhc is depleted, Khc is no longer able to transport *oskar* mRNA to the posterior pole. Consistent with this hypothesis, Khc was localized to the oocyte posterior in *luciferase* shRNA expressing oocytes, but was significantly delocalized under conditions of Dhc depletion (Figures 6A and 6B). 

A similar phenotype was also observed for the Dynactin component, Glued. Glued localized to the posterior pole and was also enriched around the oocyte nucleus in control egg chambers (Figure 6C). By contrast, Glued was no longer enriched at the posterior pole in Dhc depleted oocytes (Figure 6D). However, the perinuclear enrichment of Glued was still observed in Dhc depleted oocytes (Figure 6D). 

Based on these results, we conclude that the posterior localization of the Dynactin complex and Khc requires Dynein. Thus, under conditions of greatly reduced Dynein, Khc is unable to efficiently localize *oskar* mRNA at the posterior pole.

## Discussion

### Net-posterior localization of *oskar* mRNA requires Dynein

The localization of *oskar* mRNA at the posterior of the oocyte is essential for restricting Oskar protein to this region of the cell. Oskar protein is required for establishing the anterior-posterior polarity of the oocyte and future embryo. The localization of *oskar* mRNA is an active process that requires microtubules and the Kinesin-1 motor. Consistent with the involvement of this motor in the transport of *oskar* mRNA, Kinesin heavy chain (Khc) co-localizes with *oskar* mRNA, and loss-of-function mutants in khc result in *oskar* mRNA delocalization [18,20]. 

A paradoxical finding, however, is that Dynein heavy chain (Dhc), the motor subunit of the Dynein complex, is also enriched at the oocyte posterior [25]. A question raised by this finding is whether or not Dynein is also involved in localizing *oskar* mRNA. This has been a challenging question to answer because Dynein performs an essential function in oocyte specification. Thus, loss-of-function mutants in the Dynein complex, or in known regulators of Dynein, do not specify an oocyte [30,42,47,51]. Consequently, these mutants have not been useful in analyzing the function of Dynein during stages at which *oskar* mRNA is localized. 

In order to analyze the function of Dynein in *oskar* mRNA localization, we employed a newly described shRNA-mediated depletion strategy [43]. Using this strategy, we were able to specifically deplete Dhc in mid to late-stage egg chambers, thus bypassing the requirement for Dynein in oocyte specification. By using this approach, we provide evidence that the efficient posterior localization of *oskar* mRNA requires Dynein. 

### Nurse cell to oocyte transport

Previous results have suggested a potential function for Dynein in expediting the nurse cell to oocyte transport of localized mRNAs [33,52]. The prevailing view suggests that Dynein makes this transport step more efficient [52]. Our findings indicate that depletion of Dhc resulted in significant delocalization of *oskar* mRNA within the oocyte (Figure 3). However, we did not detect an appreciable accumulation of *oskar* mRNA in the nurse cell cytoplasm of Dhc depleted egg chambers (Figure 3). One explanation for this phenotype is that although Dynein-dependent transport rapidly delivers localized mRNAs into the oocyte, these mRNAs might also be delivered into the oocyte via cytoplasmic flows that are independent of Dynein. Alternatively, it is plausible that localization of transcripts within the oocyte is more sensitive to Dhc depletion than transport from nurse cells into the oocyte. Because our approach involves shRNA-mediated depletion, we cannot be certain that the egg chambers are completely devoid of Dhc. Any residual Dhc that might be present could be sufficient for transporting mRNAs into the oocyte, yet insufficient for specifically localizing these transcripts within the oocyte. 

### *oskar* mRNA localization within the oocyte

How might Dynein function in the posterior localization of *oskar* mRNA? One possibility is that Dynein somehow anchors the Khc/*oskar* mRNA complex at the posterior pole. An anchoring function for Dynein has been demonstrated for apically localized mRNAs in blastoderm embryos and for *gurken* mRNA in the oocyte [53,54]. 

An alternative hypothesis is that Dynein is somehow involved in the net-posterior transport of *oskar* mRNA. Our results are most consistent with this latter hypothesis. Dynein, as well as known regulators of the motor, transiently co-localized with *oskar* mRNA in wild-type oocytes. The co-localization was most obvious in stage 9 and 10a oocytes (Figure 2). However, although *oskar* mRNA remained anchored at the posterior pole in stage 10b oocytes, the posterior enrichment of Dynein was not apparent (data not shown). In addition, depletion of Dhc strongly disrupted the localization of *oskar* mRNA. However, a small enrichment of posteriorly anchored *oskar* mRNA could still be detected in these oocytes (Figures 3F’ and 3G’). Finally, disruption of the Dynactin complex, a known regulator of Dynein activity [3], caused a partial delocalization of *oskar* mRNA [32] (Figure S5). 

It is clear that Dynein by itself is unable to transport *oskar* mRNA to the posterior pole. Khc is required for posterior *oskar* mRNA localization [18]. However, under conditions of Dhc depletion, neither Khc nor *oskar* mRNA are efficiently localized at the posterior pole. What then is the function of Dynein in this context? One can envision at least three potential scenarios. In one scenario, Dynein is not present in the same complex with Khc and *oskar* mRNA, yet is somehow required for Khc activity. In another scenario, *oskar* mRNA is a dual motor cargo and is simultaneously associated with both Dynein and Khc. If this latter scenario is true, transport of *oskar* mRNA by Khc might rely on the presence of an opposite polarity motor. A mechanical strain model has been used to explain the motility of dual motor cargoes. According to this model, the mechanical strain provided by the opposite polarity motor, in this case Dynein, is required for activating the primary motor, in this case Kinesin-1 [26]. Thus, although *oskar* mRNA might not be actively transported by Dynein in wild-type oocytes, this model suggests that the mechanical activity of Dynein might still be required for the active transport of *oskar* mRNA by Khc. A final possibility is that Dynein is required for recycling Khc away from the posterior pole after it has delivered its cargo. In theory, recycling of Khc by Dynein would free up Khc for additional rounds of posterior *oskar* mRNA transport.

Future work is needed to precisely define the mechanism by which Dynein participates in localizing *oskar* mRNA at the posterior pole. 

## Materials and Methods

### Fly stocks

Unless otherwise indicated, all fly strains were grown at 25°C. Oregon-R was used as the wild-type strain. Additional fly strains used were: 

UASp-Dic-GFP; GFP-Stau (St Johnston lab); par1^w3^ [37]; par1^6323^ [37,38]; grk^2B6^ [35,36]; grk^2E12^ [14]; *dhc* shRNA-A (Bloomington stock center; #36583, donor TRiP); *dhc* shRNA-B (Bloomington stock center; #36698, donor TRiP); *luciferase* shRNA (Bloomington stock center; #35788, donor TRiP); *eb1* shRNA (Bloomington Stock center; #36680, donor TRiP); w[*]; P{w[+mC]=matalpha-GAL4-VP16}V2H (Bloomington stock center; #7062, donor Andrea Brand); P{w[+mC]=UASp-Act5C.mRFP}13, w[*] (Bloomington stock center; #24777; donor Susan Parkhurst). 

Please note that an addition maternal alpha-tubulin driver is available from the Bloomington stock center (Stock #7063). However, this driver is active in early-stage egg chambers in addition to mid and late-stage egg chambers.

The Dic-GFP transgene was constructed by cloning the dic coding sequence and 248 nt of 5’UTR upstream of the initiation codon (from clone LD22777 obtained from the *Drosophila* Genome Resource Center) into a UASp vector which allows C-terminal tagging with 3 concatemeric copies of the eGFP sequence [55]. Dic-GFP was expressed by crossing to the matalpha tubulin-Gal4 driver (stock 7062). The progeny were fattened on yeast paste for 3 days before dissection. The shRNAs were also expressed by crossing the respective strains to the matalpha tubulin-Gal4 driver. The progeny were fattened on yeast paste for 3 days prior to dissection and processing.

### Immunofluorescence

Immunofluorescence was performed as described previously [41] with a few modifications. Females fattened on yeast paste were dissected in Sf-900 II SFM (Life Technologies). The oocytes were fixed for 5 min. in PBS containing 4% formaldehyde (Pierce; catalog #28908). After fixation, the ovaries were washed in PBST (PBS containing 0.1% triton X-100), blocked and incubated with antibody. Images were captured on a Zeiss LSM 510 upright confocal microscope.

### Immunoprecipitation followed by RT-PCR

Ovaries from well-fed females were dissected in Sf-900 II SFM (Life Technologies). The ovaries were homogenized in lysis buffer A (25mM Hepes, pH 6.8, 50mM KCl, 1mM MgCl2, 1mM DTT, 125mM sucrose, 0.1% NP-40, 0.1mg/ml yeast tRNA, 0.2mg/ml salmon sperm DNA (Life Technologies), and Halt protease inhibitor cocktail (Pierce)). The lysates were cleared by centrifugation at 10,000g at 4°C for 10 min. For each immunoprecipitation, 600ug of lysate was incubated with the respective antibodies at 4°C for 1.5hrs. The complexes were isolated using proteinA coupled agarose beads (Pierce). The beads were washed four times for 5 min. each with gentle shaking at 4°C using wash buffer A (25mM Hepes, pH 6.8, 150mM KCl, 1mM MgCl2, 125mM sucrose, and 0.1% NP-40). The bound complexes were eluted in 100ul of elution buffer (100mM Hepes, pH 6.8, 150mM NaCl, 12.5mM EDTA, and 1% SDS) at 68°C for 10min. RNA was extracted from the eluate by phenol chloroform (25:24 ratio; Sigma Aldrich) extraction. The RNA pellet was resuspended in 20μl of RNAsecure (Life Technologies). Two μl of each sample was reverse transcribed using SuperscriptIII (Life Technologies) and random hexamers. *oskar*, *bicoid* and *gurken* mRNAs were examined using 35 cycles of PCR with gene specific primers. 

### Co-immunoprecipitation of protein complexes

Ovaries from well-fed females were dissected as described. The ovaries were homogenized in lysis buffer B (50mM Tris pH 7.5, 200mM NaCl, 0.2mM EDTA, 0.05% NP-40 and Halt protease inhibitor cocktail (Pierce)). The lysates were cleared by centrifugation at 10,000g at 4°C for 10min. Dic-GFP was immunoprecipitated from 600ug of total lysate using GFP-trap beads (Chromotek). The lysates were incubated with the beads for 1.5hrs. The beads were subsequently washed three times (using lysis buffer B) to eliminate non-specifically bound proteins. The bound complexes were eluted by boiling in Laemmli buffer, run on a gel, and analyzed by western blotting.

### In situ hybridization

*oskar*, *bicoid* and *gurken* mRNAs were visualized as previously described with a few modifications [41]. In brief, dissected ovaries were fixed with 4% formaldehyde for 20 mins. Next, the fixative was removed and the ovaries were washed with PBST. After wash steps, the ovaries were teased apart and incubated in a 1:1 mix of PBST:hybridization buffer (50% deionized formamide, 5x SSC, 0.1% Tween 20). This solution was then removed and pre- hybridization buffer (hybridization buffer + 50 ug/ml salmon sperm DNA), warmed to 85°C for 5 mins, was added. Pre-hybridization was performed at 65°C for 90 mins. The DIG-labeled anti-sense RNA probe was diluted in fresh hybridization buffer containing 50 ug/ml salmon sperm DNA, warmed to 85°C for 5 mins and then chilled on ice for 2 mins. Next, the pre-hybridization solution was removed, the probe was added and hybridization was performed overnight at 65°C. The next day, the samples were washed in pre-warmed hybridization buffer for 30 mins at 65°C. This solution was replaced by a 1:1 mix of PBST:hybridization buffer and incubated at 65°C for 30 mins. The samples were then washed with several changes of PBST and blocked with 2% non-fat dry milk in PBST. Next, a 1:600 dilution (in blocking solution) of peroxidase conjugated DIG antibody was added (Jackson ImmunoResearch Laboratories, Inc.). The samples were then incubated overnight at 4°C. The next day, the samples were washed with blocking solution and incubated with FITC conjugated tyramide (Perkin Elmer) diluted 1:50 in the provided amplification buffer. The amplification was performed at room temperature for 2 hrs. The samples were stained with TO-Pro3 (Life Techologies), washed with PBS and mounted in antifade. Images were captured on a Zeiss LSM 510 upright confocal microscope.

### Antibodies

Unless specifically stated, the indicated dilutions are for immunofluorescence. 

The following antibodies were used: Mouse anti-Dhc (Developmental studies hybridoma bank; 1:150; donor J. Scholey); rabbit anti-Khc (Cytoskeleton, Inc., 1:150 for immunofluorescence, 1:1500 for western); rabbit anti-GFP (Life Technologies; 1:200); rat anti-GFP (Nacalai USA, Inc.; 1:300); rabbit anti-Lis-1 (Abcam, 1:3000 for western); mouse anti-Dic (Millipore; 1:50); mouse anti-Orb (Developmental studies hybridoma bank; 1:300; donor P. Schedl); mouse anti-LaminDmO (Developmental studies hybridoma bank; clone ADL84.12; 1:100; donor P.A. Fisher); rabbit anti-Staufen (D. St Johnston; 1:2500); rabbit anti-EB1 (S. Rogers, 1:500), rabbit anti-CLIP-190 (K. Miller, 1:200), rabbit anti-Glued (V. Gelfand; 1:300 for immunofluorescence, 1:3000 for western) mouse anti-gamma-tubulin (Sigma; 1:1000 for western); rabbit anti-Osk (A. Ephrussi, 1:2000); goat anti-rabbit Alexa 594 and 488 (Life Technologies, 1:400 and 1:200 respectively); goat anti-mouse Alexa 594 (Life Technologies, 1:400); goat anti-mouse HRP (Jackson Immunoresearch; 1:5000) and goat anti-rabbit HRP (Jackson Immunoresearch; 1:5000).

## Supporting Information

Figure S1 **Dic-GFP associates specifically with *oskar* and *bicoid* mRNAs.** Ovarian lysates from flies expressing Act5c-GFP (lane 1) and Dic-GFP (lane 2) were subjected to immunoprecipitation using GFP antibody beads. The co-precipitating RNAs were extracted and analyzed using RT-PCR. *oskar* and *bicoid* mRNAs were enriched in the Dic-GFP pellet. By contrast, *vasa* and *smb* mRNAs were present at background levels in both pellets.
(EPS)

Figure S2 **Expression profile of matalpha-Gal4.** Flies containing a UASp-Act5c-mRFP transgene were crossed to the matalpha-Gal4 driver (Bloomington stock 7062). Female progeny from this cross were dissected, fixed and stained with DAPI (blue) to visualize nuclei. Act5c-RFP (red) is strongly expressed in mid to late-stage egg chambers. Upon increasing the gain, Act5c-RFP signal could also observed at lower levels in earlier stage egg chambers.
(EPS)

Figure S3 ***oskar* mRNA levels are unaffected in Dhc depleted ovaries.** Ovaries from flies expressing shRNA targeting either *luciferase* or different regions of *dhc* (*dhc* shRNA A and B) were dissected. Total RNA was extracted, and the level of *oskar* and gamma-tubulin mRNAs were determined using RT-PCR. Depletion of Dhc has no effect on the level of *oskar* mRNA.
(EPS)

Figure S4 **Depletion of EB1 does not affect Dhc and *oskar* mRNA localization.** (A-B) Ovaries from flies expressing shRNA targeting either *luciferase* or *eb1* were dissected and processed for immunofluorescence using an antibody against EB1. EB1 was abundantly expressed in the germline of control oocytes, but was significantly depleted in egg chambers expressing shRNA targeting *eb1*.
(C) Ovaries from flies expressing shRNA targeting *eb1* were processed for in situ hybridization using probes against *oskar* mRNA. No defects were observed in the localization of oskar mRNA.
(D) Ovaries from flies expressing shRNA targeting *eb1* were processed for immunofluorescence using an antibody against Dhc. Dhc was enriched at the posterior pole in EB1 depleted oocytes.
(EPS)

Figure S5 **Over-expression of p50/Dynamitin delocalizes *oskar* mRNA.** (A) Ovaries from wild-type flies were processed for in situ hybridization using anti-sense probes against *oskar* mRNA.
(B-C) Ovaries from flies expressing UASp-p50/Dmn driven using the maternal alpha-tubulin driver, were processed for in situ hybridization using anti-sense probes against *oskar* mRNA. The level of posteriorly localized *oskar* mRNA was decreased upon p50 over-expression (B). In parallel, the level of delocalized *oskar* mRNA within the oocyte was increased in these egg chambers (C). Panel C represents the same egg chamber depicted in ‘B’ imaged using increased gain.
(EPS)
