# Correction: Dynein Associates with *oskar* mRNPs and Is Required For Their Efficient Net Plus-End Localization in *Drosophila* Oocytes

**DOI:** 10.1371/annotation/1d5f32a4-54f6-4633-a8b4-f0fae0627507

**Published:** 2014-01-03

**Authors:** Paulomi Sanghavi, Shobha Laxani, Xuan Li, Simon L. Bullock, Graydon B. Gonsalvez

In the Funding Statement, the name of a funding organization was given incorrectly. The Funding Statement should read:

"Graydon B. Gonsalvez was supported by grants from the American Cancer Society (123887-RSG-13-006-01-CCG) and the National Institutes of Health (1R01GM100088-01A1). Simon Bullock was supported by MRC core funding (U105178790) and a Lister Institute Research Prize. The funders had no role in study design, data collection and analysis, decision to publish, or preparation of the manuscript." 

